# Sudanese pearl millet (*Pennisetum glaucum* (L.) R. Br.) germplasm reveals genetic potential for carotenoid improvement and provitamin a biofortification

**DOI:** 10.1038/s41598-026-45956-6

**Published:** 2026-03-25

**Authors:** Khitma A. Sir Elkhatim, Nikwan Shariatipour, Manhal Gobara Hamid, Faraz Muneer, Mohammed Elsafy, Mahbubjon Rahmatov, Tilal Abedlhalim

**Affiliations:** 1 Biotechnology and Biosafety Research Center, Agricultural Research Corporation, Shambat, Khartoum, North Sudan; 2 College of Agricultural Studies, University of Science and Technology, Shambat, Khartoum, North Sudan; 3 Department of Plant Breeding, Swedish University of Agricultural Sciences (SLU), PO Box 190, Alnarp, 234 22 Sweden; 4 Geo-Biosphere Interactions, Department of Geosciences, University of Tuebingen, Schnarrenbergstr. 94-96, 72076 Tuebingen, Germany

**Keywords:** Hidden hunger, Carotenoids, Genetic variation, Biofortification, Multivariate statistics, Biochemistry, Biotechnology, Genetics, Plant sciences

## Abstract

Pearl millet is a vital staple in Sudan’s arid and semi-arid regions and offers a promising platform for biofortification to alleviate provitamin A deficiency. This study evaluated the genetic variation in carotenoid content and grain color attributes of 116 Sudanese pearl millet accessions under field conditions at the Al Gezira Research Station. Carotenoid profiling (β-carotene, lutein, zeaxanthin, and total carotenoids) was performed using spectrophotometry and high-performance liquid chromatography (HPLC). Grain color traits were assessed using a Chroma Meter and CIE-LAB color space parameters. Highly significant differences were observed among the genotypes (*P* < 0.0001) across all traits. β-carotene ranged from 0.028 to 0.763 µg/g, lutein from 0.11 to 4.79 µg/g, and zeaxanthin from 0.05 to 1.69 µg/g, with total carotenoids reaching up to 9.15 µg/g. Grain color attributes, such as L*, a*, b*, ΔE, and BI, exhibited substantial variability, with strong correlations among b*, ΔE, and the browning index (BI). Broad-sense heritability estimates were high for all traits (> 94%), with β-carotene, lutein, and zeaxanthin exhibiting high genetic advancement as a percentage of the mean (GAM > 140%), indicating a strong selection potential. Stepwise regression identified lutein and a* as the major predictors of variation (R² > 11%). Cluster and principal component analyses revealed distinct groups of carotenoid-dense genotypes, with HSD12345, HSD12415, and HSD12516 ranking among the top for β-carotene content, respectively. The identified high-carotenoid accessions and color-linked traits provide valuable resources for biofortification and breeding programs to improve the nutritional quality of pearl millet in drought-prone regions.

## Introduction

Pearl millet (*Pennisetum glaucum* (L.) R. Br.) is a crucial cereal crop extensively cultivated in the rural and arid regions of South Asia and sub-Saharan Africa, including Sudan, where it serves as a staple food for millions of people. Sudan is recognized as the center of origin and genetic diversity of pearl millet^1^, hosting a rich reservoir of landraces and wild relatives that offer substantial potential for crop improvement. These landraces are known for their exceptional nutritional value and remarkable adaptability to harsh environmental conditions, such as poor soils and erratic rainfall^2^. The inherent drought tolerance of pearl millet, combined with its ability to thrive under high-temperature stress, makes it a strategic crop for enhancing food and nutritional security in climate-vulnerable regions^3^.

In Sudan, particularly in the Darfur and Kordofan regions, pearl millet is the primary food source for most of the inhabitants. It is predominantly cultivated to prepare a wide array of traditional dishes that are deeply embedded in the local food culture, such as stiff porridge (“*Asida*”), thin fermented flatbread (“*Kisra*”), thin gruel (“*Nasha*”), nutrient-rich paste (“*Medida*”), non-alcoholic fermented drink (“*Abreih*”), and traditional beverages like (“*Marisa*”)^4^. These diverse food applications not only reflect the cultural significance of pearl millet but also highlight its culinary versatility and year-round consumption in rural households.

Pearl millet grains are rich in complex carbohydrates, high-quality proteins, dietary fiber, and antioxidants, all of which contribute to overall health and prevent chronic diseases^5^. They also contain elevated levels of essential micronutrients, including iron (Fe), zinc (Zn), calcium (Ca), potassium (K), magnesium (Mg), and manganese (Mn), as well as essential vitamins such as thiamine (B1) and niacin (B3), and amino acids such as lysine and tryptophan^6–8^.

Despite its nutritional richness and cultural importance, PM remains underutilized in the national dietary strategies. However, its inherent potential to address micronutrient deficiencies renders it relevant to public health efforts, particularly in food-insecure regions^6,8^. Micronutrient malnutrition, particularly vitamin A deficiency (VAD), remains a widespread global health concern^9^. VAD manifests as night blindness, xerophthalmia, weakened immune function, and increased vulnerability to infections such as measles and diarrhea^10^. These effects are especially severe during critical life stages characterized by heightened nutritional demands, including early childhood, pregnancy, and lactation. VAD affects an estimated 190 million preschool-aged children and 20 million pregnant women, significantly contributing to immunodeficiency, stunted growth, and elevated mortality rates^11^.

The World Health Organization (WHO) has identified Sudan as one of the 46 countries with high prevalence rates of VAD, with the burden most concentrated in low-income and food-insecure communities across sub-Saharan Africa and Southeast Asia^12^. The carotenoid content of pearl millet, a widely consumed staple crop, urgently requires improvement, given the persistence of VAD and its devastating health consequences. Biofortification through conventional breeding and molecular approaches is a promising, sustainable, and cost-effective strategy for enhancing provitamin A levels in local food systems and mitigating the public health burden of micronutrient malnutrition.

To address the high prevalence of vitamin A deficiency (VAD), attention has increasingly focused on carotenoids, which are naturally occurring yellow to red fat-soluble pigments found in higher plants and cereal grains that serve as dietary precursors of vitamin A. They are classified into two major classes: hydroxylated xanthophylls (lutein, zeaxanthin, and α-cryptoxanthin) and hydrocarbons (α-carotene, β-carotene, and lycopene)^13^. In pearl millet, the carotenoid profile is primarily characterized by lutein and zeaxanthin as the dominant xanthophylls, together with β-carotene as the principal provitamin^14–16^. Among the more than 600 carotenoid compounds identified in plants, α-carotene, β-carotene, and α-cryptoxanthin are nutritionally significant because of their provitamin A activity^17^.

Biofortification efforts often aim to enhance β-carotene accumulation by redirecting metabolic flux within the carotenoid biosynthesis pathway or upregulating key genes involved in the early biosynthetic steps. This approach has been successfully applied to several staple crops to improve the provitamin A content without compromising agronomic performance^18^. Previous research has largely focused on total carotenoid and β-carotene content, with substantial genetic variation reported for these traits. However, information on the genetic diversity of individual xanthophylls remains scarce. In this context, the genetic richness of Sudanese pearl millet landraces, particularly those from the Greater Darfur and Kordofan regions, offers valuable opportunities for carotenoid biofortification^14^. To the best of our knowledge, no prior studies have documented genetic variation in lutein and zeaxanthin in pearl millet grains, making this study the first to characterize their variation across diverse germplasms. By quantifying the grain carotenoid profiles of these diverse landraces and using modern breeding techniques, biofortified pearl millet varieties capable of reducing provitamin A malnutrition could be developed^19^.

This study aimed to quantify the concentrations of the key carotenoids lutein, zeaxanthin, and β-carotene in a diverse panel of Sudanese pearl millet landraces and identify genotypes with elevated carotenoid contents for potential use in biofortification breeding programs targeting vitamin A deficiency in Sudan and comparable dryland regions.

## Materials and methods

### Plant materials and field experiment

The field experiment was conducted at the Gezira Research Station Farm, located in Wad Medani, Al Gezira State, Sudan (latitude 14°24′ N, longitude 33°29′ E; altitude 406.9 m above sea level), approximately 210 km south of Khartoum. The site is characterized by heavily cracked clay soils with an alkaline pH (8.3).

A genetically diverse panel comprising 115 Sudanese pearl millet accessions and one improved cultivar (Supplementary Table S1) was obtained from the Agricultural Plant Genetic Resources Conservation and Research Center (APGRC) of the Agricultural Research Corporation (ARC) in Sudan. Most accessions originated from the pearl millet-growing regions of western Sudan.

The experiment was conducted using a randomized complete block design (RCBD) with three replicates per accession during the 2022 rainy season. Standard agronomic practices were followed to ensure optimal crop growth. Before panicle emergence, grains were harvested from ten controlled, self-pollinated plants per accession. The harvested grains were then carefully dried, cleaned, and purified by manually removing foreign materials, broken or damaged kernels, and visibly discolored grains. The samples were milled and sieved through a 0.4-mm mesh to obtain fine pearl millet flour, which was stored in sealed polypropylene bags at − 20 °C for up to four weeks for carotenoid content analysis.

### Carotenoid extraction

Following the preparation and storage of the pearl millet flour samples, carotenoid extraction was performed as a distinct preliminary step for subsequent quantification and chromatographic analysis. Given the high sensitivity of carotenoids to light, oxygen, and temperature, all extractions were conducted under yellow light conditions (below 50 lx, achieved by shielding ambient laboratory light) and at − 2 °C to minimise oxidative and photo-induced degradation, thereby preserving carotenoid integrity. Extraction was performed solely to isolate carotenoids from the flour matrix without quantification at this stage. Effective extraction and appropriate solvent selection are critical prerequisites for reliable carotenoid analysis^20^. Total carotenoids were extracted using a liquid–liquid extraction method adapted from Jacques et al.^21^, and extracts intended for individual carotenoid analysis, including β-carotene, lutein, and zeaxanthin, were prepared using a hot saponification protocol based on Fratianni et al.^22^. The extracts were processed immediately for downstream quantification or chromatographic separation.

### Quantification of total carotenoids

Total carotenoid quantification was performed independently of chromatographic analysis, following the protocol described by Jacques et al.^21^, with minor modifications to optimize the extraction from pearl millet flour. Specifically, 2 g of finely milled pearl millet flour from each accession was mixed with 25 mL of acetone and ultrasonicated for 10 min at 20 °C to enhance pigment release. The mixture was filtered through Whatman No. 1 filter paper to obtain a clear supernatant. The filtrate was transferred into a separating funnel and partitioned with 20 mL of petroleum ether. To remove residual acetone, the solution was washed with distilled water (100 mL). The aqueous acetone layer was discarded, and the fractionation process was repeated twice to ensure complete extraction of the phenolic compounds.

The resulting petroleum ether phase was passed through a Whatman No. 1 filter paper containing 5 g of anhydrous sodium sulfate to eliminate moisture. The clarified extract was directly quantified using a UV–Vis spectrophotometer, with the absorbance measured at 450 nm. The total carotenoid content was calculated as micrograms of β-carotene equivalents per gram of dry matter using Eq. 1 (Eq. 1):

1 $$\:\mathrm{T}\mathrm{o}\mathrm{t}\mathrm{a}\mathrm{l}\:\mathrm{c}\mathrm{a}\mathrm{r}\mathrm{o}\mathrm{t}\mathrm{e}\mathrm{n}\mathrm{o}\mathrm{i}\mathrm{d}\mathrm{s}\:\:=\left(\frac{\mathrm{A}\:\mathrm{x}\:\mathrm{V}\:\mathrm{x}\:{10}^{4}}{{\mathrm{E}}_{1\mathrm{\%}\:}\mathrm{x}\:\mathrm{P}}\right)$$

Where *A is the* absorbance at 450 nm, *V* is the total volume of the extract, *P* is the sample weight (g), and *E*_*1*_*%*^*1*^ *cm* is the extinction coefficient of β-carotene in petroleum ether (2592).

### Carotenoid profile using high-performance liquid chromatography

The extraction of individual carotenoids for chromatographic profiling was conducted separately from total carotenoid quantification. Individual carotenoids (β-carotene, lutein, and zeaxanthin) were extracted using a modified hot saponification protocol based on Fratianni et al.^22^, which was optimized for pearl millet flour. Briefly, 100 mg of the milled sample was placed in a microcentrifuge tube and subjected to hot saponification at 158 °C for 30 min, with gentle mixing every 10 min.

The sample was homogenized using 250 µL of ethanol containing pyrogallol (60 g/L), 100 µL of ethanol (95%), 100 µL of sodium chloride solution (10 g/L), and 100 µL of potassium hydroxide solution (600 g/L). Following saponification, carotenoids were extracted twice with 750 µL each of a sodium chloride solution and an n-hexane/ethyl acetate (9:1) mixture. The phases were then centrifuged at 1500 rpm for 5 min. The organic (upper) phase was collected and evaporated to dryness under reduced pressure. The dried residue was reconstituted solely for chromatographic separation and identification, as described below.

### Carotenoid profiling by HPLC

Following reconstitution, individual carotenoid compounds were separated, identified, and purified using normal-phase high-performance liquid chromatography (HPLC), as described by Hussain et al.,^23^, with modifications adapted from Panfili et al.^24^. Carotenoids were separated using a Phenomenex LUNA Silica column (250 × 4.6 mm i.d., 5 μm particle size). The mobile phase consisted of n-hexane with 5% isopropyl alcohol and was delivered at a constant flow rate of 1.5 mL/min. Detection was performed using a fluorescence detector set at 450 nm, with an injection volume of 100 µL per sample. To ensure analytical precision and reproducibility, the column was cleaned with 10% isopropyl alcohol in n-hexane (v/v) after every 12 injections. The carotenoid compounds in the samples were identified by comparing their retention times and absorption spectra with those of the authenticated standards.

### Grain color measurement

A Chroma Meter CR-400 (Konica Minolta, Japan) was used to characterize the pericarp color of each pearl millet genotype. Before measurement, the instrument was calibrated using a standard white reflector plate to ensure accuracy. Grain color was quantified using the CIE-LAB color space, which defines three primary color parameters: L* (lightness), a* (position on the green-to-red axis), and b* (position on the blue-to-yellow axis). These primary color values were used to evaluate the grain appearance and visual quality^25^.

To provide a more comprehensive characterization of grain color, additional derived indices were calculated, including the total color difference (ΔE) (ΔΕ, Eq. 2), whiteness index (WI, Eq. 3), saturation index (SI, Eq. 4), the hue angle (Hue, Eq. 5), total color difference, and BI ( Eq. Six and 7), according to the “CIE-LAB” colorimetric system (Eq. 2):

2 $$\:{\Delta\:}{{\rm\:E}}\:=\sqrt{{({L}_{0}-{L}^{*}{)}^{2}+({a}_{0}-{a}^{*})}^{2}+({b}_{0}{-{b}^{*})}^{2}}$$

In this context, the subscript “0” indicates the color reading of the standard white reflector plate used as the reference.

3 $$\:\mathrm{W}\mathrm{I}\hspace{0.17em}=\hspace{0.17em}100\:-\sqrt{{(100-{L}^{*})}^{2}+{{a}^{*}}^{2}+{{b}^{*}}^{2}}$$

4 $$\:\mathrm{S}\mathrm{I}\:=\sqrt{{{a}^{*}}^{2}+{{b}^{*}}^{2}}$$

5 $$\:\mathrm{H}\mathrm{u}\mathrm{e}\hspace{0.17em}=\hspace{0.17em}\mathrm{a}\mathrm{r}\mathrm{c}\mathrm{t}\mathrm{a}\mathrm{n}\left(\:\raisebox{1ex}{${b}^{*}$}\!\left/\:\!\raisebox{-1ex}{${a}^{*}$}\right.\:\right)$$

6 $$\:\mathrm{B}\mathrm{I}\:=\frac{100(x-0.31)}{0.17}$$

Where *x*,

7 $$\:\mathrm{x}\:=\frac{({a}^{*}+1.75{L}^{*})}{(5.645{L}^{*}+{a}^{*}-0.012\:{b}^{*})}$$

### Statistical and biometrical-genetic analyses

To assess genetic variability among the tested pearl millet genotypes, an analysis of variance (ANOVA) was performed using a randomized complete block design (RCBD) to determine the significance of genotypic effects on carotenoid and grain color traits. Variance components were estimated using a general linear model (GLM) in which the genotype effects were treated as fixed. The expected mean squares were used to calculate the genetic parameters, as outlined in Table 2.

The phenotypic variance ($\:{{\upsigma\:}}_{P}^{2}$), genotypic variance ($\:{{\upsigma\:}}_{\mathrm{g}}^{2}$) and environmental variance ($\:{{\upsigma\:}}_{\mathrm{e}}^{2}$) were estimated following the method of Federer et al.^26^, using the following equations:

8 $$\:{{\upsigma\:}}_{\mathrm{g}}^{2}=\frac{{\mathrm{M}\mathrm{S}}_{\mathrm{g}}-{\mathrm{M}\mathrm{S}}_{\mathrm{e}}}{\mathrm{r}}$$

9 $$\:{{\upsigma\:}}_{\mathrm{e}}^{2}={\mathrm{M}\mathrm{S}}_{\mathrm{e}}$$

10 $$\:{{\upsigma\:}}_{\mathrm{P}}^{2}={{\upsigma\:}}_{\mathrm{g}}^{2}+{{\upsigma\:}}_{\mathrm{e}}^{2}$$

The phenotypic (PCV) and genotypic (GCV) coefficients of variation were calculated according to Singh and Chaudhary^27^ and Burton and DeVane^28^ using the mean (µ) of the population as a scaling factor (Eqs. 11 and 12):

11 $$\:\mathrm{P}\mathrm{C}\mathrm{V}=\frac{\sqrt{{\sigma\:}_{p}^{2}}}{\mu\:}\times\:100$$

12 $$\:\mathrm{G}\mathrm{C}\mathrm{V}=\frac{\sqrt{{\sigma\:}_{g}^{2}}}{\mu\:}\times\:100$$

where $\:{\upmu\:}$ is the mean of the tested traits.

Broad-sense heritability (H²) was calculated using the formula proposed by Lush^29^(Eq. 13):

13 $$\:{\mathrm{H}}^{2}=\frac{{{\upsigma\:}}_{\mathrm{g}}^{2}}{{{\upsigma\:}}_{\mathrm{p}}^{2}}$$

Genetic advance (GA) and genetic advance as a percentage of the mean (GAM) were calculated according to Burton^30^ and Johnson et al.^31^ and (Eqs. 14 and 15):

14 $$\:\mathrm{G}\mathrm{A}={\mathrm{k}\times\:{\upsigma\:}}_{\mathrm{e}}\times\:\frac{{\mathrm{H}}^{2}}{100}$$

In this context, k denotes the standardized selection differential (selection intensity), with a value of 2.063 at a 5% selection proportion; σₑ denotes the phenotypic standard deviation of the trait; and H² indicates the broad-sense heritability.

15 $$\:\mathrm{G}\mathrm{A}\mathrm{M}=\frac{\mathrm{G}\mathrm{A}}{{\upmu\:}}\times\:100$$

Correlation coefficients were estimated between the traits. Phenotypic and genotypic correlation coefficients were calculated according to Singh and Chaudhary^27^ from the variance components estimated based on the expected mean squares in the ANOVA (Eqs. 16 and 17):

16 $$\:{\mathrm{r}}_{\mathrm{p}\left(\mathrm{X}\mathrm{Y}\right)}=\frac{{\mathrm{S}}_{\mathrm{p}\left(\mathrm{X}\mathrm{Y}\right)}}{{\mathrm{S}}_{\mathrm{p}\left(\mathrm{X}\right)}\times\:{\mathrm{S}}_{\mathrm{p}\left(\mathrm{Y}\right)}}$$

17 $$\:{\mathrm{r}}_{\mathrm{g}\left(\mathrm{X}\mathrm{Y}\right)}=\frac{{\mathrm{S}}_{\mathrm{g}\left(\mathrm{X}\mathrm{Y}\right)}}{{\mathrm{S}}_{\mathrm{g}\left(\mathrm{X}\right)}\times\:{\mathrm{S}}_{\mathrm{g}\left(\mathrm{Y}\right)}}$$

Where, the $\:{\mathrm{r}}_{\mathrm{p}\left(\mathrm{X}\mathrm{Y}\right)}$, $\:{\mathrm{S}}_{\mathrm{p}\left(\mathrm{X}\mathrm{Y}\right)}$, $\:{\mathrm{S}}_{\mathrm{p}\left(\mathrm{X}\right)}$, $\:{\mathrm{S}}_{\mathrm{p}\left(\mathrm{Y}\right)}$, $\:{\mathrm{r}}_{\mathrm{g}\left(\mathrm{X}\mathrm{Y}\right)}$, $\:{\mathrm{S}}_{\mathrm{g}\left(\mathrm{X}\mathrm{Y}\right)}$, $\:{\mathrm{S}}_{\mathrm{g}\left(\mathrm{X}\right)}$, and $\:{\mathrm{S}}_{\mathrm{g}\left(\mathrm{Y}\right)}$ are the phenotypic correlation between traits X and Y, phenotypic covariance between traits X and Y, root of the phenotypic variance of trait X, root of the phenotypic variance of trait Y, the genotypic correlation between traits X and Y, genotypic covariance between traits X and Y, root of the genotypic variance of trait X and root of the genotypic variance of trait Y, respectively. Stepwise regression was used to examine the variability in β-carotene levels^32^. A heat map was constructed using the Canberra distance metric and Ward’s D2 linkage algorithm to visualize patterns of similarity among pearl millet genotypes and identify groups with comparable carotenoid and agronomic trait profiles. Statistical analyses were performed using SAS version 9.4 (SAS Institute, Cary, NC, USA) for descriptive statistics and analysis of variance, and the R packages Variability^33^ to estimate genetic parameters, corrplot^34^ to visualize pairwise correlations among traits, and pheatmap^35^ to generate clustered heatmaps illustrating multivariate relationships among genotypes and measured variables.

## Results

### Carotenoid composition across pearl millet accessions

The results of the analysis of variance (ANOVA) revealed highly significant genotypic differences among 116 Sudanese pearl millet accessions for all measured carotenoid traits, including β-carotene, lutein, zeaxanthin, and total carotenoids, as well as for the full suite of grain color attributes (Table 1).

**Table 1:** Analysis of variance (ANOVA) for carotenoid profiles and grain color attributes in 116 pearl millet accessions.

Source of variation	DF	ß-Carotene	Lutein	Zeaxanthin	Total Carotenoids	L*	a*	EMS
Block	2	1.34E-04	1.62E-04	7.16E-05	0.019	1.192	0.010	$\:-$
Genotype	115	0.099^***^	2.177^***^	0.281^***^	6.784^***^	14.818^***^	0.697^***^	$\:{\sigma\:}_{e}^{2}+{r\sigma\:}_{g}^{2}$
Residual	230	3.67E-05	2.01E-04	1.56E-05	0.048	0.308	4.33E-03	$\:{\sigma\:}_{e}^{2}$
CV		2.52	1.18	0.89	6.14	0.66	16.95	
Source of variation	DF	b*	∆E	WI	BI	SI	Hue	EMS
Block	2	0.218	0.017	0.066	0.013	0.217	0.252	$\:-$
Genotype	115	4.452^***^	8.416^***^	9.831^***^	21.146^***^	4.424^***^	4.316^***^	$\:{\sigma\:}_{e}^{2}+{r\sigma\:}_{g}^{2}$
Residual	230	0.023	0.069	0.097	0.132	0.023	0.083	$\:{\sigma\:}_{e}^{2}$
CV		0.65	1.11	0.43	1.17	0.43	30.10	

DF: degree of freedom; L*: brightness; a*: redness; b*: yellowness; ∆E: total color difference; WI: whiteness index; BI: browning index; SI: saturation index; Hue: hue angle; G: genotype; r: replication; $\:{\sigma\:}_{e}^{2}$: error variance; $\:{\sigma\:}_{g}^{2}$: genotypic variance; EMS: expected mean square; *** represent significant at *P* < 0.0001, respectively.

For β-carotene, the genotypic mean square was 0.099. Thewhich significantly exceeded the residual mean square (3.67 × 10⁵), and the coefficient of variation (CV) was low (2.52%), indicating high experimental precision. Similarly, both lutein and zeaxanthin displayed highly significant genotypic effects, with genotype mean squares of 2.177 and 0.281, respectively, and correspondingly low error variances. The CVs for these traits (1.18% for lutein and 0.89% for zeaxanthin) further confirmed the reliability of trait measurements and experimental control. The analysis of total carotenoids was equally robust, with a highly significant genotype effect (mean square of 6.784) and a moderate CV of 6.14%, which is acceptable for biochemical traits such as pigment concentration (Table 1).

**Fig. 1:**
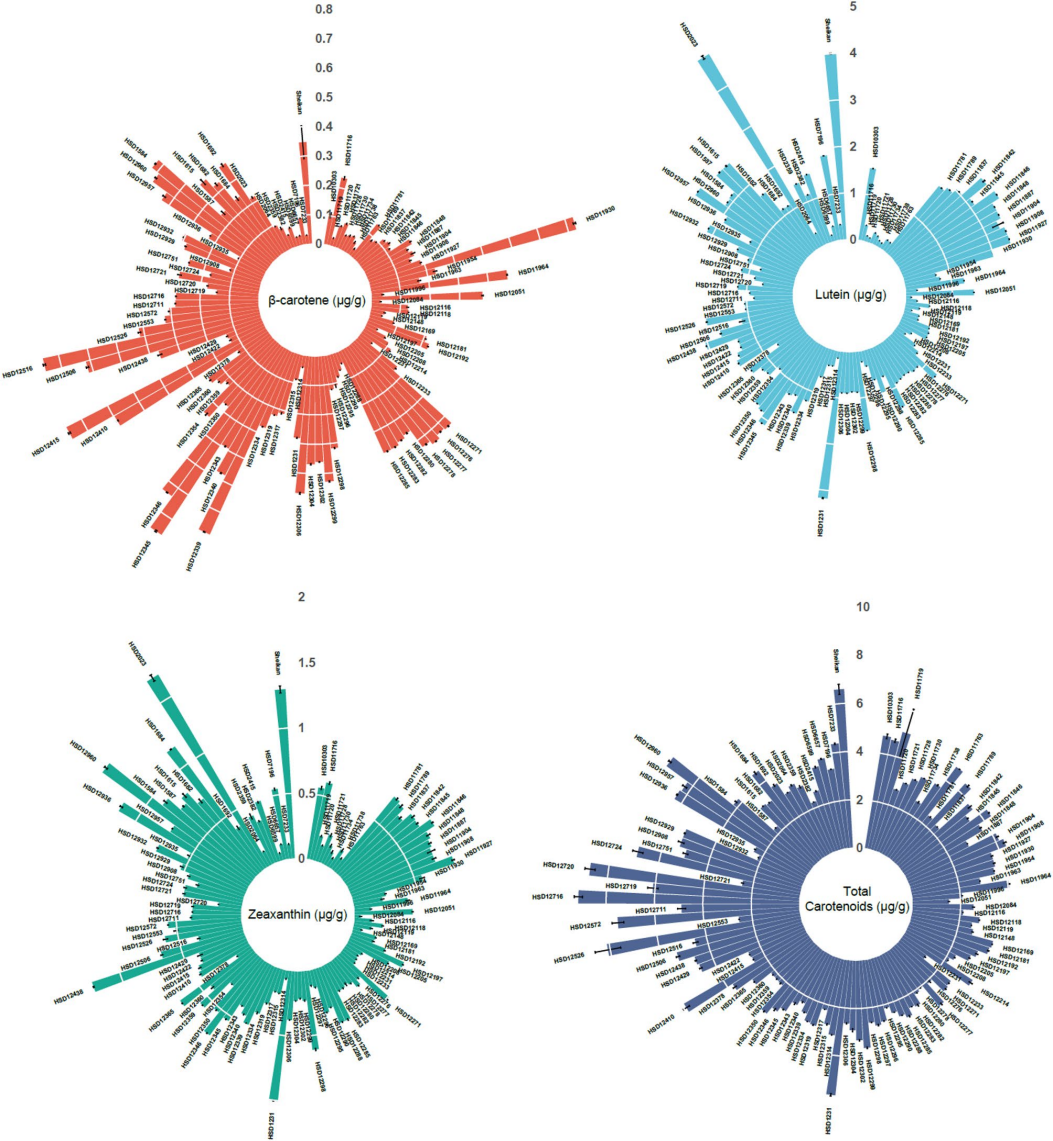
Mean values of carotenoid profiles of 116 pearl millet accessions.

The β-carotene content, the most nutritionally significant provitamin A compound, ranged from 0.028 µg/g in HSD11719 to 0.763 µg/g in HSD12345, indicating a nearly 27-fold difference between the lowest and highest accessions (Fig. 1, Supplementary Table S2). Several accessions, such as *HSD12415* (0.758 µg/g), *HSD12516* (0.754 µg/g), and *HSD11930* (0.727 µg/g), had higher levels, indicating that they are strong candidates for provitamin.

Lutein, a key xanthophyll with eye health-promoting properties, varied from 0.106 µg/g (*HSD11720*) to 4.786 µg/g (HSD2023), demonstrating an even broader range of variation. Notably, *HSD1231*, *Sheikan*, and *HSD12957* recorded lutein values above 2.7 µg/g, highlighting their relevance as multi-carotenoid donors. Similarly, the zeaxanthin content ranged from 0.048 µg/g in HSD12084 to 1.693 µg/g in HSD2023, reinforcing the exceptional carotenoid density of this accession. Other genotypes, such as *HSD12960*, *HSD12438*, and *HSD12936*, also accumulated high levels of zeaxanthin (> 1.0 µg/g) (Fig. 1, Supplementary Table S2). The total carotenoid content varied from 1.273 µg/g in HSD12231 to 9.146 µg/g in HSD12716, followed by *HSD12526* (8.095 µg/g), *HSD12720* (8.755 µg/g), and *HSD12572* (7.538 µg/g).

### Colorimetric traits among sudanese pearl millet genotypes

The analysis also revealed significant genotypic variation in grain color attributes (Table 1). Lightness (L*) and redness (a*) showed significant genotype effects, with a robust measurement of lightness (L*) and a CV of 0.66%. The moderately higher CV for a* (16.95%) indicated more natural variation, as expected for pigment-related traits. Yellowness (b*) exhibited a genotype mean square of 4.452 and a notably low CV of 0.65%, indicating consistent and reliable differentiation across the tested pearl millet accessions.

Furthermore, the derived colorimetric indices, including the total color difference (∆E), whiteness index (WI), browning index (BI), saturation index (SI), and hue angle (hue), exhibited highly significant genotype effects. The mean squares for these traits ranged from 4.316 to 21.146, whereas the CVs remained low (0.43%-1.17%) for all traits except for hue angle, which had a CV of 30.10%.

The grain lightness (L*) values across the 116 Sudanese pearl millet genotypes ranged from 79.34 (HSD2064) to 88.83 (HSD12304). Several genotypes exhibited exceptionally high L* values, including HSD12304 (88.83), HSD12711 (88.57), HSD12116 (88.37), and HSD12280 (88.49), indicating very light-colored grains. In contrast, genotypes HSD2064 (79.34%), HSD11904 (80.01%), and HSD1231 (80.46%) showed darker grain appearances. The least significant difference for L* (LSD) at *P* < 0.05 for L* was 0.89, highlighting the robust capacity to distinguish between genotypic differences in lightness (Supplementary Table 2).

For the a* parameter, the values ranged from strongly negative (green hues) to slightly positive (red hues). The most negative a* values were recorded for genotypes HSD12084 (–1.517), HSD11738 (–1.487), and HSD12116 (–1.420), indicating strong greenish hues. In contrast, positive a* values, reflecting reddish hues, were observed in genotypes such as HSD12285 (0.910), HSD12908 (0.603), HSD12506 (0.600), and HSD12929 (0.450). The LSD for a* was 0.11, providing a clear resolution for detecting variations in redness and greenness among the accessions and ensuring measurement accuracy (Supplementary Table 2).

The yellowness (b*) values ranged from 19.4426.45 to. The highest b* values were recorded for genotypes HSD2023 (26.45), HSD12936 (25.68), HSD11963 (25.48), and HSD12306 (25.81), whereas the lowest were recorded for HSD6599 (19.44) and HSD12084 (20.16). The total color difference (ΔE) ranged from 19.8027 to 41. The highest ∆E values were observed for HSD2064 (27.41), HSD12306 (27.26), and HSD12936 (26.68), whereas HSD12116 (19.80), HSD12084 (20.22), and HSD11738 (20.63) showed the lowest values (Supplementary Table 2).

The genotypes of the whiteness index (WI) ranged from 67.61 to 75.95. The highest WI was measured in HSD12116 (75.95), HSD11738 (75.17), and HSD12084 (75.05), while the lowest was observed in HSD2064 (67.61), HSD12306 (67.96), and HSD11963 (68.32). The browning index (BI) ranged from 24.7137 to 08. Genotypes HSD12306 (37.08), HSD12936 (36.63), and HSD11963 (36.39) had the highest BI, while HSD12084 (24.71) and HSD12116 (25.04) recorded the lowest (Supplementary Table 2).

The saturation index (SI) varied from 19.4626 to 48. The most saturated colors were found in HSD2023 (26.48), HSD12936 (25.68), and HSD11963 (25.48), while the lowest SI values were observed in HSD6599 (19.46) and HSD12084 (20.22). The hue angle (hue) ranged from − 1.57 to 1.57°, with most genotypes exhibiting slightly negative values. A few genotypes, such as HSD11837 (1.57), HSD11781 (1.56), and HSD12936 (1.56), exhibited the highest positive hue values, while HSD12169, HSD12339, and HSD12340 showed the lowest values at -1.570 (Supplementary Table 2).

### Genetic parameters of carotenoid profile and grain color traits in pearl millet

The mean β-carotene content was 0.24 µg/g, with a minimum of 0.03 and a maximum of 0.181. It exhibited a genotypic coefficient of variation (GCV) of 75.43%, phenotypic coefficient of variation (PCV) of 75.47%, and broad-sense heritability (H²) of 0.9989. The genetic advance (GA) was 0.37, and the genetic advance as a percentage of the mean (GAM) was 155.53% (Table 2).

Lutein had a mean concentration of 1.20 µg/g, ranging from 0.10 to 0.850. The GCV and PCV were 71.14% and 71.15%, respectively, indicating high heritability. The value was 0.9997, with GA and GAM values of 1.76 and 146.74%, respectively. Zeaxanthin had a mean of 0.44 µg/g, with a minimum of 0.05 µg/g and a maximum of 0.305 µg/g. GCV and PCV were 69.15% and 69.16%, respectively, whereas heritability reached 0.9998. GA and GAM values were 0.63 and 142.65%, respectively (Table 2). The total carotenoid content had a mean value of 3.57 µg/g, with a minimum of 1.23 µg/g and a maximum of 1.51 µg/g. GCV was 41.96%, PCV was 42.40%, and heritability was 0.9791. GA was estimated to be 3.06, and GAM was 85.64% (Table 2).

**Table 2:** Mean values, phenotypic coefficients of variation (PCV), genotypic coefficients of variation (GCV), broad-sense heritability (H^2^), genetic advance (GA), and genetic advance as a percentage of the mean (GAM) of the carotenoid profile and grain color attributes were assessed in 116 accessions of pearl millet.

Trait	Mean ± SE	Max	Min	GCV (%)	PCV (%)	H^2^	GA	GAM (%)
ß-Carotene	0.24 ± 0.004	0.03	0.181	75.43	75.47	0.9989	0.37	155.53
Lutein	1.20 ± 0.008	0.10	0.850	71.14	71.15	0.9997	1.76	146.74
Zeaxanthin	0.44 ± 0.002	0.05	0.305	69.15	69.16	0.9998	0.63	142.65
Total Carotenoids	3.57 ± 0.127	1.23	1.510	41.96	42.40	0.9791	3.06	85.64
L*	84.44 ± 0.320	78.18	2.263	2.60	2.69	0.9402	4.40	5.21
a*	-0.39 ± 0.038	-1.53	0.484	123.74	124.90	0.9816	0.98	252.92
b*	23.39 ± 0.088	19.43	1.221	5.19	5.23	0.9845	2.49	10.63
∆E	23.58 ± 0.152	19.75	1.684	7.07	7.16	0.9758	3.40	14.41
WI	71.84 ± 0.180	65.73	1.823	2.51	2.54	0.9709	3.66	5.10
BI	31.13 ± 0.210	24.66	2.664	8.50	8.58	0.9815	5.41	17.37
SI	23.40 ± 0.088	19.45	1.218	5.18	5.22	0.9844	2.48	10.59
Hue	-0.96 ± 0.167	-1.57	1.219	123.99	127.59	0.9442	2.38	248.55

L*: brightness, a*: redness, b*: yellowness, ∆E: Total color difference, WI: Whiteness index, BI: Browning index, SI: Saturation index, Hue: Hue angle, SE: Standard Error of the Mean.

For grain color attributes, L* (brightness) showed a mean of 84.44, with values ranging from 78.18 to 2.263. GCV, PCV, heritability, GA, and GAM were 2.60%, 2.69%, 0.9402, 4.40, and 5.21%. The a* (red-green) component had a mean of − 0.39, a range from − 1.53 to 0.484, and recorded GCV and PCV values of 123.74% and 124.90%, respectively. The heritability was 0.9816, whereas GA and GAM were 0.98 and 252.92%, respectively (Table 2).

The b* (yellowness) component recorded a mean of 23.39, with a GCV of 5.19%, and a PCV of 5.23%. The heritability was 0.9845, and GA and GAM were 2.49 and 10.63%, respectively. The mean total color differences (∆E) were 23.58, 7.07, 7.16, 0.9758, 3.40, and 14.41%, respectively. The whiteness index (WI) averaged 71.84, with a GCV of 2.51%, PCV of 2.54%, heritability of 0.9709, GA of 3.66, and GAM of 5.10%. The browning index (BI) had a mean of 31.13, GCV of 8.50%, PCV of 8.58%, heritability of 0.9815, GA of 5.41, and GAM of 17.37% (Table 2).

The saturation index (SI) had a mean of 23.40, with a GCV of 5.18%, PCV of 5.22%, heritability of 0.9844, GA of 2.48, and GAM of 10.59%. The mean hue angle was − 0.96, with values ranging from − 1.57 to 1.219. GCV, PCV, heritability, GA, and GAM were 123.99%, 127.59%, 0.9442, 2.38, and 248.55%, respectively (Table 2).

### Correlation between carotenoid traits and grain color attributes

**Fig. 2:**
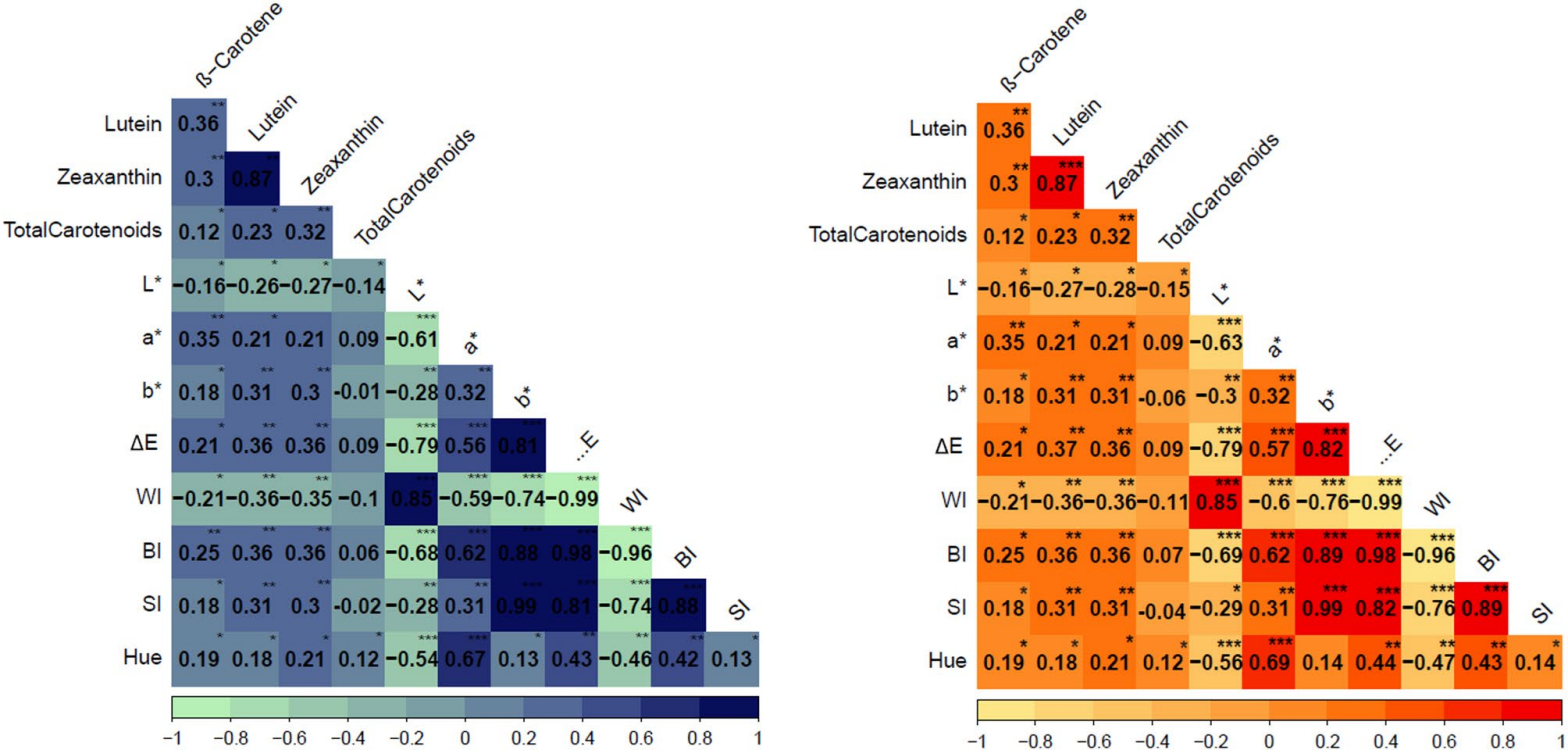
Phenotypic (rp) and genotypic (rg) correlation coefficients for carotenoid traits and grain color attributes in 116 pearl millet accessions.

As shown in Fig. 2, correlation analysis revealed strong and significant associations between several carotenoid and grain traits. Among the carotenoids, lutein and zeaxanthin were highly correlated at both phenotypic and genotypic levels (rp = 0.87 and rg = 0.87, respectively). β-Carotene showed a weak but positive correlation with total carotenoids (rp = 0.12, rg = 0.12) and moderate correlations with lutein (rp = 0.36, rg = 0.36) and zeaxanthin (rp = 0.30, rg = 0.30).

Among the grain color attributes, strong correlations were observed between b* and the saturation index (SI) (rp = 0.99, rg = 0.99), browning index (BI) (rp = 0.88, rg = 0.89), and total color difference (ΔE) (rp = 0.81, rg = 0.82). Similarly, ΔE exhibited a strong positive correlation with the BI (rp = 0.98, rg = 0.98) and SI (rp = 0.81, rg = 0.82). Lightness (L*) correlated positively with the whiteness index (WI) (rp = 0.85, rg = 0.85) but negatively with ΔE (rp = -0.79, rg = -0.79), BI (rp = -0.68, rg = -0.69), and redness (a*) (rp = -0.61, rg = -0.63). The strongest negative correlations were observed between WI and ΔE (rp = -0.99, rg = -0.99) and WI and BI (rp = -0.96, rg = -0.96).

Among the carotenoid and color traits, a* exhibited the highest positive correlation with β-carotene (rp = 0.35, rg = 0.35). In contrast, L* (rp = -0.16, rg = -0.16) and WI (rp = -0.21, rg = -0.21) showed weak negative associations with β-carotene.

### Stepwise regression analysis between grain color traits and β- Carotene content

**Fig. 3:**
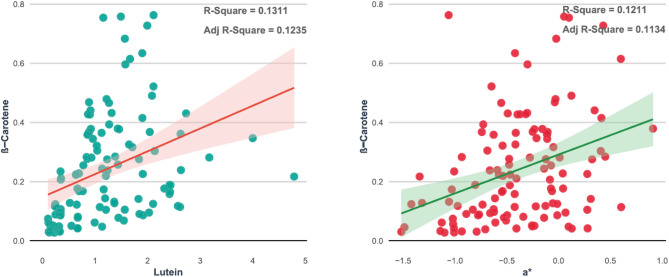
Stepwise regression analysis between lutein and a* traits with ß-carotene content in 116 pearl millet accessions.

Stepwise regression analysis was performed to identify the grain color parameters that significantly predicted β-carotene content in 116 pearl millet accessions (Fig. 3; Table 3). Among the predictors, lutein exhibited a positive and significant relationship with β-carotene, explaining approximately 13.11% of the total variation (R² = 0.1311; Adj R² = 0.1235; *F* = 17.20;*p* < 0.001). Similarly, the redness parameter *(a)* also showed a significant positive association with β-carotene content, accounting for 12.11% of the variation (R² = 0.1211; Adj R² = 0.1134; *F* = 15.70, *p < 0.001). The positive slope coefficients (0.077 for lutein and 0.131 for a) suggest that both traits improve β-carotene levels in pearl millet breeding programs.

**Table 3:** Results of stepwise regression analysis between grain color and ß-carotene content in pearl millet accessions.

ß-Carotene				
Variable entered	Parameter estimate	R^2^	Adj R^2^	F Value
Lutein	0.077	0.1311	0.1235	17.20***
a*	0.131	0.1211	0.1134	15.70***

### Multivariate analysis, represented by principal component analysis (PCA), included a heatmap

**Fig. 4:**
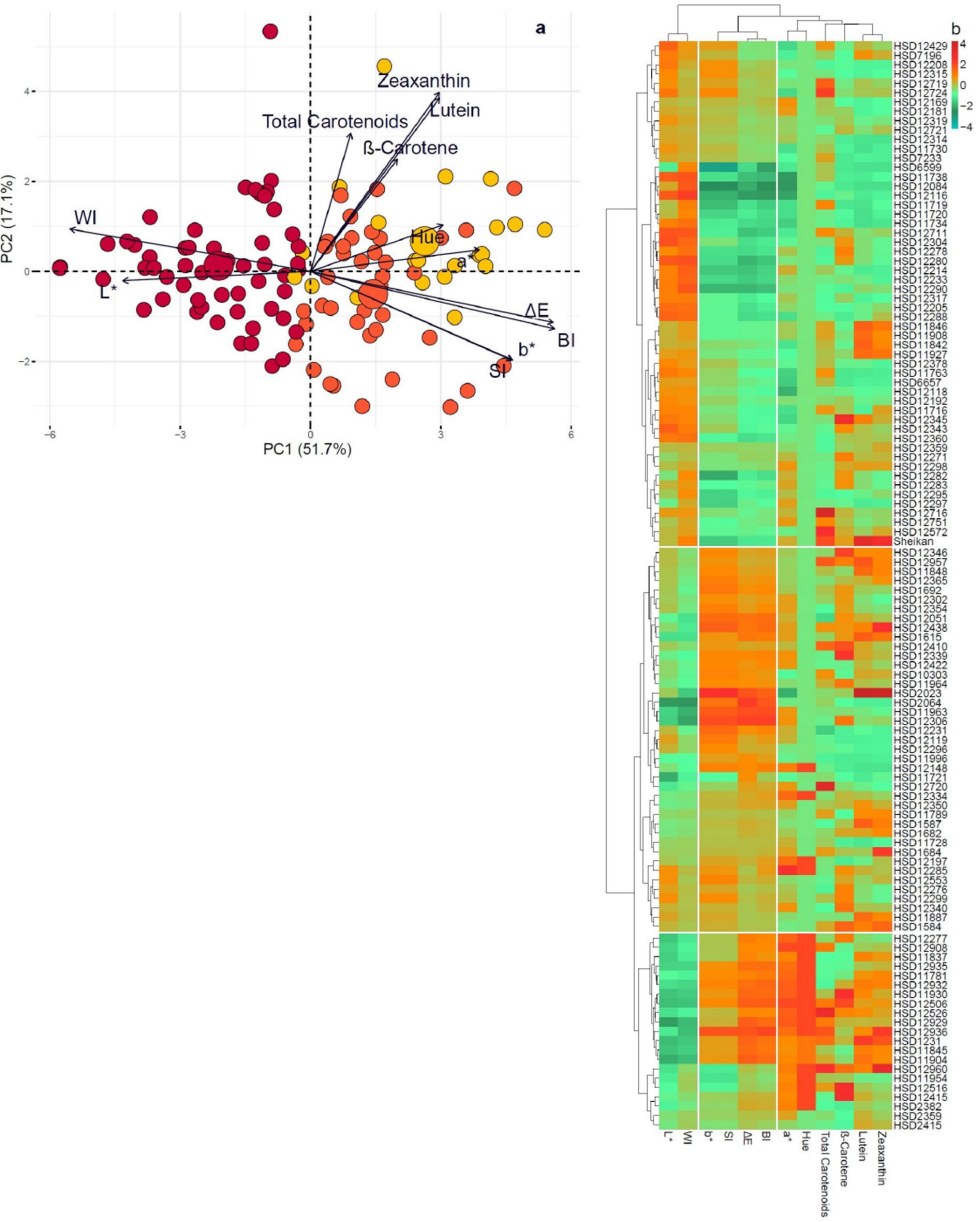
Projection of the first (PC1) and second (PC2) genotypic principal components (**a**) and two-dimensional heatmap dendrogram (**b**) of 116 pearl millet accessions tested for carotenoid and grain color traits. Dendrograms illustrate the relationships between accessions (rows) and traits (columns) based on variations in the shade color. L*, brightness; a*, redness; b*, yellowness; ∆E: Total color difference; WI: Whiteness index; BI: Browning index; SI: Saturation index; Hue: Hue angle.

PCA results revealed that the first two principal components (PCs) accounted for 51.7% and 17.1% of the total variance, respectively. The biplot revealed that carotenoid traits (β-carotene, lutein, zeaxanthin, and total carotenoids) clustered tightly and were positively associated with the positive sides of PC1 and PC2. Among these, the zeaxanthin and lutein vectors showed a strong alignment, indicating a high degree of correlation. Similarly, β-carotene and total carotenoids were closely associated, suggesting their co-expression. In contrast, the grain-color parameters exhibited distinct spatial patterns. The chromaticity parameters b*, ∆E, hue, and BI were positively correlated with PC1, whereas lightness (L*) and the whiteness index (WI) were strongly associated with the negative side of PC1. The a* and SI parameters displayed a moderate association, projecting diagonally onto the positive PC1 quadrant (Fig. 4a).

A two-dimensional hierarchical clustered heatmap revealed distinct clustering patterns, with higher carotenoid content in accessions HSD12716, HSD12345, and HSD12415, which formed distinct clusters characterized by elevated levels of zeaxanthin, lutein, β-carotene, and total carotenoids (indicated by red shading) (Fig. 4b). These accessions also exhibited high b* and hue values, consistent with deeper yellow and orange pigmentation. In contrast, accessions with high WI and L* values, such as HSD12106 and HSD12173, clustered together, indicating lighter white-to-creamy grain phenotypes with lower carotenoid content (green shading). Traits such as BI, SI, and ∆E exhibited moderate variability across the dataset, contributing to the distinctions between the subgroups (Fig. 4b).

### Carotenoid-dense

Table 4 presents the top ten pearl millet accessions identified as germplasm with high carotenoid content based on their superior carotenoid profiles. For β-carotene, accessions HSD12345, HSD12415, and HSD12516 exhibited high concentrations of 0.49–0.76 µg/g. The highest lutein content was recorded in the HSD2023 and Sheikan accessions, with values ranging from 2.43 to 4.79 µg/g. Zeaxanthin levels ranged from 0.83 to 1.69 µg/g, with HSD2023, HSD12960, and Sheikan ranking highest. The total carotenoid content was highest in HSD12716, HSD12720, and HSD12526, with values ranging from 6.09 to 9.15 µg/g.

**Table 4:** Top 10 core collection pearl millet accessions with carotenoid traits and grain color attributes.

Trait	Class	Top 10 accessions	Range
ß-Carotene (µg/g)	High	HSD12345, HSD12415, HSD12516, HSD11930, HSD12339, HSD12346, HSD12506, HSD12410, HSD1584, HSD12960	0.49–0.76
Lutein (µg/g)	High	HSD2023, Sheikan, HSD1231, HSD12957, HSD1587, HSD1615, HSD11846, HSD11842, HSD11848, HSD11887	2.43–4.79
Zeaxanthin (µ	High	HSD2023, HSD12960, Sheikan, HSD12438, HSD1684, HSD12936, HSD1615, HSD11927	0.83–1.69
Total Carotenoids (µg/g)	High	HSD12716, HSD12720, HSD12526, HSD12572, HSD12960, HSD12724, Sheikan, HSD12936, HSD12957, HSD12719	6.09–9.15

## Discussion

Pearl millet is increasingly recognized for its climate resilience and nutritional value, particularly its high carotenoid content. Among these, β-carotene plays a central role in biofortification strategies to alleviate vitamin A deficiency, a major public health concern in many developing countries, including Sudan^17^. In line with the study objectives, this study examined the extent of carotenoid diversity, the biofortification potential of Sudanese germplasm, and the feasibility of grain color traits as indirect selection tools, which are particularly relevant for low-input agricultural systems in sub-Saharan Africa, where conventional supplementation and fortification approaches are often unsustainable^6,8^.

In line with the first objective, we quantified the variation in key carotenoids (β-carotene, lutein, and zeaxanthin) across a diverse panel of Sudanese pearl millet accessions using high-performance liquid chromatography (HPLC). The β-carotene content among the 116 accessions ranged from 0.028 to 0.763 µg/g, with accessions HSD12345, HSD12415, and HSD12516 emerging as the leading candidates for provitamin A enrichment. These levels notably exceeded those previously reported by Bashir et al.^14^, who observed a narrower β-carotene range (0.01–0.126 µg/g) in 225 Sudanese pearl millet landraces. However, our findings were lower than the broader variability (0.46–2.83 µg/g) reported by Sathya et al.^15^ in Indian germplasm, suggesting regional differences in genetic potential and environmental influences.

Lutein and zeaxanthin, two non-provitamin A xanthophylls essential for visual health and oxidative stress mitigation^36^, also showed substantial genotypic variation. Importantly, this study provides the first evidence that these xanthophylls are genetically variable in pearl millet, extending the current knowledge beyond total carotenoids and β-carotene. The consistent predominance of lutein over zeaxanthin mirrors the patterns reported in foxtail millet^37^, suggesting conserved biosynthetic regulation across millet species and opening opportunities for comparative genomics and cross-species trait discovery.

Our findings establish a foundation for developing pearl millet cultivars biofortified with lutein and zeaxanthin, with potential benefits for visual health and mitigation of oxidative stress, particularly in populations vulnerable to micronutrient deficiencies. The consistent predominance of lutein over zeaxanthin across genotypes provides clear guidance for trait prioritization in breeding programs. This biochemical diversity can be effectively exploited through selection and hybridization, supported by marker-assisted breeding. When integrated with evaluations of correlations with yield, maturity, and grain quality, these strategies will enable the development of climate-resilient, nutritionally enhanced cultivars. Collectively, these advances strengthen food system resilience and improve public health and nutrition in regions where pearl millet is a dietary staple.

Based on the observed genetic diversity, the identification of accessions with superior carotenoid profiles directly addressed the second objective of this study. The total carotenoid content of the Sudanese pearl millet accessions exhibited substantial genotypic variation, ranging from 1.273 µg/g in HSD12231 to 9.146 µg/g in HSD12716. Several other accessions, including HSD12526 (8.095 µg/g), HSD12720 (8.755 µg/g), and HSD12572 (7.538 µg/g), demonstrated consistently high carotenoid levels. These accessions represent potential high-carotenoid-content germplasm rather than a defined core collection and constitute valuable genetic resources for biofortification breeding.

Our findings are consistent with those of Khangura et al.^38^, who identified the parental lines PIB 128 and Pb 111 B as superior combiners for β-carotene, total carotenoids, grain yield, and hardness, highlighting the feasibility of integrating nutrient-dense traits into breeding programs. The agreement between these earlier GCA analyses and our current germplasm evaluation reinforces the effectiveness of pedigree-based selection and hybrid breeding in achieving simultaneous gains in both nutritional and agronomic traits. Accordingly, incorporating Sudanese pearl millet accessions with high total carotenoid content into breeding pipelines represents a promising strategy for developing climate-resilient, nutrient-enriched pearl millet cultivars to reduce provitamin A deficiencies in vulnerable populations.

A key objective of this study was to assess whether grain color traits could serve as a cost-effective and breeder-friendly proxy for carotenoid selection, particularly in low-input agricultural systems in sub-Saharan Africa^6,8^. Our findings revealed a significant positive correlation between β-carotene content and the CIE a* color parameter (rp = rg = 0.35) and a negative correlation between β-carotene content and both lightness (L*) and whiteness index (WI), indicating that darker and redder grains tend to be enriched in provitamin A carotenoids. Similar relationships have been reported in wheat and triticale^39^, demonstrating the broader applicability of colorimetric traits as phenotypic indicators.

In contrast to the findings in sorghum^40^, no significant correlation was detected between grain yellowness (b *) and carotenoid content. In sorghum, grain yellowness effectively discriminated between accessions with and without carotenoids, and luminosity (L*) was a stronger predictor of carotenoid concentration within yellow grain groups. Several grain color genes (e.g., *ZEP*, *CYP97A*, and *LycE*) have also been reported to co-localize with carotenoid QTLs. Pigment biosynthesis pathways and a comparatively narrow range of grain pigments.

Importantly, although grain color traits such as a* and L* show partial associations with carotenoid levels in pearl millet, their predictive power remains limited, particularly for discriminating variations among similarly colored grains. Consequently, while visual or digital colorimetry may serve as a cost-effective tool for early-stage screening, it cannot replace biochemical quantification (e.g., HPLC) in breeding programs aimed at the precise enhancement of provitamin A. Integrating color-based assessments with targeted carotenoid profiling may accelerate pre-breeding selection in resource-limited environments. In contrast, direct quantification remains essential for downstream selection and varietal release. Future research should explore the genomic associations between grain-color traits and carotenoid biosynthetic genes in pearl millet, drawing on insights from the sorghum model to improve the precision of marker-assisted selection.

Beyond diversity and proxy-based selection, our results revealed exceptionally high broad-sense heritability (H² ≥ 99%) and genetic advance as a percentage of the mean (GAM > 140%) for both lutein and zeaxanthin, indicating strong additive genetic control and high selection efficiency under classical pedigree-based breeding. These parameters suggest that substantial genetic gains can be achieved through routine selection, without extensive environmental buffering. This strong genetic control is consistent with the findings of Cruet-Burgos et al.^41^, who reported moderate heritability for zeaxanthin (61.2%) and lutein (56.9%) in a global sorghum panel, despite pronounced genotype-by-environment (G × E) effects. The higher estimates observed here likely reflect tighter genetic control within the Sudanese pearl millet germplasm evaluated under relatively uniform field conditions, whereas the sorghum panel encompassed broader agroecological variation. Nevertheless, both studies converge to demonstrate that lutein and zeaxanthin are readily amenable to genetic improvement.

Moreover, the strong phenotypic and genotypic correlations between lutein and zeaxanthin (rp = 0.87; rg = 0.87), consistent with those reported by Cruet-Burgos et al.^41^, support the feasibility of co-selection strategies in breeding programs. Such trait covariation simplifies the construction of selection indices and facilitates the pyramiding of multiple nutritional traits into elite cultivars. Collectively, these findings underscore the value of integrating carotenoid profiling into mainstream breeding pipelines, where high heritability, favorable trait interdependence, and manageable G × E effects can accelerate the development of micronutrient-enriched cultivars tailored to the needs of nutritionally vulnerable populations in dryland regions.

## Conclusion

This study demonstrated substantial genetic variation in carotenoid content among Sudanese pearl millet accessions, with elite genotypes such as HSD12716 and HSD12526 exhibiting high levels of total carotenoids, β-carotene, lutein, and zeaxanthin. The exceptionally high broad-sense heritability (H² > 99%) and genetic advance (GAM > 140%) indicate strong additive genetic control, confirming that these traits are highly amenable to improvement through conventional pedigree-based selection. Positive associations between β-carotene and grain color attributes, particularly a*, further support the use of grain color as a cost-effective screening tool in the early breeding stages, although biochemical quantification remains essential for the precise selection of high-β-carotene genotypes.

Despite the strength of these findings, carotenoid evaluation was conducted in a single environment, and trait expression may vary under contrasting climatic conditions. The absence of genomic marker data also limits direct inferences of the genetic architecture. Nevertheless, the identification of high-carotenoid donor lines, combined with strong genetic control and favorable trait relationships, provides a robust foundation for the pearl. Future efforts should prioritize multi-environment testing and the integration of GWAS and genomic prediction to enhance the efficiency of selection. Targeted crossing of high-carotenoid accessions with high-yielding, farmer-preferred varieties provide a practical pathway for developing nutritionally enhanced, climate-resilient pearl millet cultivars, thereby reducing micronutrient deficiencies among vulnerable dryland populations.

## Data Availability

All data supporting the findings of this study are presented in the manuscript and its supplementary materials. The details of the pearl millet accessions evaluated in this study are provided in Supplementary Table S1. Supplementary Table S2 summarizes the mean values of carotenoid profiles and grain color attributes across all accessions.
